# Editorial: Therapeutic approaches in youth psychiatry – The art of balancing between ‘do not harm’ and ‘best attainable care’

**DOI:** 10.3389/fpsyt.2023.1137997

**Published:** 2023-02-10

**Authors:** Soumitra Das, Alby Elias, Sheikh Shoib, Barikar C. Malathesh, Adesh Kumar Agrawal, Thilini Jayasooriya

**Affiliations:** ^1^Department of Psychiatry, NorthWestern Mental Health, Parkville, VIC, Australia; ^2^Royal Melbourne Hospital, Melbourne, VIC, Australia; ^3^Department of Psychiatry, The University of Melbourne, Parkville, VIC, Australia; ^4^Department of Psychiatry, Director of Health Services Srinagar, Srinagar, India; ^5^Department of Psychiatry, All India Institute of Medical Sciences Bibinagar, Hyderabad, Telangana, India; ^6^Department of Psychiatry, National Institute of Mental Health and Neurosciences (NIMHANS), Bangalore, Karnataka, India

**Keywords:** youth, therapy, ethics, risk, harm and benefits

The developmental trajectories in youth are unique. Many youths have a mix of developmental traits from children and adults. The youth age, usually considered from 10 to 24 years, are influenced by social changes and ever-growing attempts to control such changes. The period can be uncertain due to ongoing sexual maturity, lack of financial and housing stability, and dependency on others yet trying to be independent. Youth age is also considered emergency adulthood as most experience dynamic qualities of love or relationship, work, and education where there is perceived stability in adulthood. As per the concept, the five main features of a youth developmental phase are identity exploration, instability, self-focus, feeling in between, possibilities and optimism (1).

It is the most vulnerable age to develop most psychiatric disorders. According to the Great Smoky Mountain study, around 61% have a diagnosable mental health issue, and 21% have impairment by the age of 21 years. The study was based on cumulative evidence from 1,420 participants from 9 through 21 years (2). Identity exploration can be exciting; however, one can be confused about their identity and develop severe anxiety or mood disorder. Instability can lead to social isolation, overreliance on social media and lack of access to the healthcare system. Excessive self-focus can lead to social exclusion and worsening of depression or disordered eating at an early age. Encouraging health optimism can help develop resilience, which can work as a protective factor in preventing long-term consequences of mental health issues. So, an early intervention approach can avert long-term social, legal and educational drawbacks (1, 3).

In our topic, we tried to emphasize the need for balancing acts while treating youth mental health issues. The young brain is still not fully matured, so they have a higher risk of developing iatrogenic problems. The first experience with mental health systems often influences their later engagement with the services. Over and under-diagnosis can be detrimental to a youth's typical development trajectory. It is not uncommon to see misdiagnosis of several mental health issues such as psychosis, mood disorder or ADHD in adolescence (4). Most of the time, the presentations are still in the prodromal phase, which may or may not develop into a full-blown illness. It is still contentious whether to treat someone with the idea of prevention where the evidence for the prevention strategies is not that great. Hence, it falls in the clinical and team approach to make a shared decision with the youth individual (5).

In this section, we have received various papers that emphasize the influences of social media and the experience of war on youth mental health. Social media is another world for youth. On the one hand, they develop social relations, which helps in skill development; on the other, it also influences different pathological behavior. An article by Fremer et al. shows the influences of social media in developing Tourette-like behavior. Another study by Razjouyan et al. discuss the war experiences among schoolgoers. It shows the importance of sociocultural stability and family supports as protective factors. In the survey, girls student suffers more from daily stressors leading to depression and anxiety.

Another paper from the section by Woodberry et al. proposes a newer model based on self-guided knowledge. The model “SCREEN–TRIAGE–ENGAGE” tries to reduce too much on the provider's expertise and focuses on a collaborative model. A person can learn about mental health issues from a designated website and engage with the clinician to explore further. In the end, the section also brings the need for exploring the efficacy of tDCS as a non-invasive intervention for depressive, anxiety and conduct disorders in adolescents (Konicar et al.). Non-invasive interventions such as rTMS or tDCS have significantly improved psychiatric care due to their excellent side effects profile and increased acceptance. The efficacy of such therapies in different disorders is still being investigated, with good results in depression. However, further studies must establish its safety and effectiveness in youth settings.

To conclude, mental health develops in social and cultural milieu. Therefore, societal norms conducive for mental health development are crucial for healthy trajectories. This is particularly important given the influence of social learning (6). The first step to a balanced approach in youth mental health is to understand the biopsychosocial formulation of the development of various disorders in this age group. A developmental framework would be beneficial while assessing and managing a youth individual. There is a need to develop safer biological and psychological treatment strategies to reduce iatrogenic harm and provide the best possible care.

## Author contributions

SD and AE have critically evaluated the papers and written the editorial. All the authors were involved in the discussion of the suitability of each submission to the journal. SS, BM, AA, and TJ modified and helped in the final paper. All authors contributed to the article and approved the submitted version.

## References

[1] Arnett JJ Žukauskiene R Sugimura K. The new life stage of emerging adulthood at ages 18-29 years: implications for mental health. Lancet Psychiatry. (2014) 1:569–76. 10.1016/S2215-0366(14)00080-7 26361316

[2] Copeland W Shanahan L Costello EJ Angold A. Cumulative prevalence of psychiatric disorders by young adulthood: a prospective cohort analysis from the Great Smoky Mountains Study. J Am Acad Child Adolesc Psychiatry. (2011) 50:252–61. 10.1016/j.jaac.2010.12.014 21334565 PMC3049293

[3] McGorry PD Goldstone SD Parker AG Rickwood DJ Hickie IB. Cultures for mental health care of young people: an Australian blueprint for reform. Lancet Psychiatry. (2014) 1:559–68. 10.1016/S2215-0366(14)00082-0 26361315

[4] Offidani E Fava GA Sonino N. Iatrogenic comorbidity in childhood and adolescence: new insights from the use of antidepressant drugs. CNS Drugs. (2014) 28:769–74. 10.1007/s40263-014-0184-0 24980773

[5] Raven M Stuart GW Jureidini J. ‘Prodromal' diagnosis of psychosis: Ethical problems in research and clinical practice. Austr New Zeal J Psychiatry. (2012) 46:64–5. 10.1177/0004867411428917 22247095

[6] Zimmerman BJ. Social learning, cognition, and personality development. In:Smelser NJ Baltes PB, editors. International Encyclopedia of the Social & Behavioral Sciences. Oxford: Pergamon (2001). p. 14341–5. 10.1016/B0-08-043076-7/01765-4

